# Scaling behavior for electric vehicle chargers and road map to addressing the infrastructure gap

**DOI:** 10.1093/pnasnexus/pgad341

**Published:** 2023-10-25

**Authors:** Alexius Wadell, Matthew Guttenberg, Christopher P Kempes, Venkatasubramanian Viswanathan

**Affiliations:** Department of Mechanical Engineering, Carnegie Mellon University, Pittsburgh, PA 15213, USA; Department of Mechanical Engineering, University of Michigan, Ann Arbor, MI 48109, USA; Department of Mechanical Engineering, Carnegie Mellon University, Pittsburgh, PA 15213, USA; Department of Mechanical Engineering, University of Michigan, Ann Arbor, MI 48109, USA; The Santa Fe Institute, Santa Fe, NM 87501, USA; Department of Mechanical Engineering, Carnegie Mellon University, Pittsburgh, PA 15213, USA; Department of Mechanical Engineering, University of Michigan, Ann Arbor, MI 48109, USA

**Keywords:** electric vehicles, infrastructure, scaling

## Abstract

Enabling widespread electric vehicle (EV) adoption requires a substantial build-out of charging infrastructure in the coming decade. We formulate the charging infrastructure needs as a scaling analysis problem and use it to estimate the EV infrastructure needs of the USA at a county-level resolution. We find that gasoline and EV charging stations scale sub-linearly with their respective vehicle registrations, recovering the sub-linear scaling typical of infrastructure. Surprisingly, we find that EV charging stations scale super-linearly with population size within counties, deviating from the sub-linear scaling of gasoline stations. We discuss how this demonstrates the infancy of both EVs and EV infrastructure while providing a framework for estimating future EV infrastructure demands. By considering the power delivery of existing gasoline stations, and appropriate EV efficiencies, we estimate the EV infrastructure gap at the county level, providing a road map for future EV infrastructure expansion.

Consumer interest in Electric Vehicles (EV) has been rising as EVs approach price parity with internal combustion engine (ICE) vehicles. However, the lack of sufficient Electric Vehicle Supply Equipment (EVSE), popularly called charging stations, could slow future adoption (1–4). Thus, identifying the placement of EVSE to meet this growing demand is essential and has been an area of extensive research (1–3, 5, 6). Prior work in the area has relied predominantly on mathematical optimization to maximize captured vehicle flow, minimize infrastructure costs, or account for power grid-related issues (2). Bottom-up optimization-based methods can precisely place and size EVSE based on the local demand and peculiarities. However, they can become challenging and computationally prohibitive for larger-scale analysis as a complex system of trade-offs emerges, which brings into question which factors are feasible to include and what form the loss function should take (1, 2).

On the other hand, a coarse-grained scaling analysis approach provides a tractable way to assess the infrastructure needs of large regions. In particular, scaling relationships demonstrate how features change with system size and often illustrate that a single set of mechanisms is governing a system (7–9). Even when the specific mechanisms are unidentified, the coarse-grained regularities of a system, as captured by the scaling exponents, often provide a powerful foundation for building models, making forecasts, and interpreting dominant trade-offs (7–12), while avoiding the computational costs and data requirements of existing methods (1, 2, 5). Here we formulate the EVSE infrastructure problem in a scaling analysis framework $\left(Y=Y_{0}N^{\beta }\right)$, which connects the EVSE infrastructure needs (*Y*) for a particular region to its population size (*N*) via the scaling exponent (*β*) and pre-factor ($Y_{0}$). While originally motivated by examples from biology (10) later works have connected urban scaling theory to conservation laws (7), evolutionary constraints (8), and economic production functions (12).

Sub-linear scaling (β<1) results in *decreasing* per capita rates with population size as agglomeration effects drive increased efficiency (10). For example, the length of a city’s road network grows sub-linearly with its population as the hierarchical structure of freeways, arterial, and local roads efficiently connect residents and aid future expansion (7). Prior works have found sub-linear scaling in the number of post offices (13), the number of libraries (11), and the funding of community colleges (9); all systems where future expansion is supported by prior efforts. Conversely, super-linear scaling (β>1) results in *increasing* per capita rates with population size; and is typical for systems that rely on social interactions or provide increasing returns with size (7, 10). For example, personal income has been shown to scale super-linearly with population (7, 10, 12), as well as, museum usage (11), and funding for research universities (9).

We find that EVSE infrastructure scales sub-linearly with EV registrations and with a scaling exponent similar to gasoline stations (Fig. 1a); suggesting that charging stations mirror the scaling behavior of gasoline stations. However, EVSE infrastructure scales super-linearly with the county population (Fig. 1c); the indirect network effects linking EV adoption and EVSE infrastructure (4). We expect the present super-linear scaling with population to be a transient phenomenon. As EVs become broadly adopted, we expect EVSE infrastructure to trend towards the sub-linear scaling currently demonstrated by the EV registration data and other infrastructures.

**Fig. 1.**
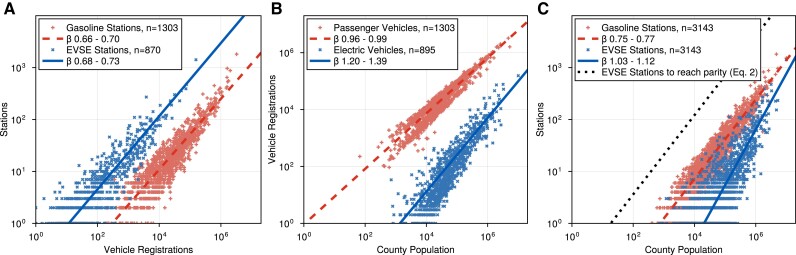
EVSE and gasoline stations scaling applied to different populations. a) EVSE and gas stations scale sub-linearly, following economies of scale, with their respective vehicle registrations per county. b) Passenger vehicle registrations scale linearly with the county population, while EV registration scales super linearly with the county population. c) Gasoline stations scale sub-linearly with the county population, while EVSE scale super-linearly with the county population; suggesting the influence of wealth/social interactions. As EV adoption increases, we expect the EV registration with county population scaling to become linear, matching the corresponding passenger vehicle registration scaling, which will drive the scaling of EVSE with county population sub-linear. Using Eq. 2, we predict that 2 million EVSE stations are needed, assuming all ports deliver $P_{EVSE}=400$ kW and all charging occurs at public charging stations (γ=1). Presently, no county has sufficient charging infrastructure, and the median county needs less than 250 new stations to reach power parity. Figure legends report the 95% confidence intervals for *β* and the number of observations used for each fit.

Finally, we leverage the eventual scaling exponent of EVSE stations to forecast future infrastructure needs. To our knowledge, this is the first application of scaling analysis to forecast the future infrastructure demands of emerging technology. Our application here to EVSEs opens up a variety of future work in scaling theory.

##  

### Urban scaling for infrastructure

We expect both gasoline and EVSE stations to behave like infrastructure assets and thus scale sub-linearly (β≈0.8) with population (7, 10). To verify this, we curated a dataset of EVSE stations, EV registrations, gasoline stations, passenger vehicle registrations, and population size at the county level; allowing the application of urban scaling both with respect to the overall population (Fig. 1c) as well as with the subgroup utilizing the refueling/recharging infrastructure (Fig. 1a). We then fit various models following the procedure developed by Leito et al. (11). We found strong evidence for power-law scaling over alternative models using this procedure. As shown in Fig. 1a, both EVSE (β=0.70±0.03) and gasoline (β=0.68±0.02) stations scale sub-linearly with their respective population. We repeated this procedure using Core-Based Statistical Areas, instead of counties, as the spatial unit of the analysis. The results are consistent with our analysis presented here and are presented in our Supplementary Material.

As explored by Bettencourt (7), sub-linear scaling reflects the incremental growth of infrastructure networks as they gradually expand to connect more people. Additionally, the decreasing number of stations per vehicle is consistent with the increasing competition between stations as EVSE infrastructure expands (4). Sub-linear scaling reflects the increasing efficiencies of agglomeration, driven in part by a more competitive market consistent with economic models (4, 12).

#### Scaling with population

Urban scaling describes how cities scale in response to individuals’ average needs or production output (7, 10). In Fig. 1a, we examined the scaling of gasoline and EVSE stations with their respective vehicle registrations, effectively focusing on the slice of the population that utilized refueling/charging infrastructure. The near ubiquity of passenger vehicles in America results in linear scaling with population size (Fig. 1b). These vehicles are supported by refueling infrastructure that scales sub-linearly, as competition between stations encourages increasing efficiencies with size (Fig. 1c); consistent with standard spatial competition models from locational economics (4). Conversely, both EVs (Fig. 1b) and EVSE infrastructure (Fig. 1c) display larger scaling exponents than their conventional counterparts, suggesting a consolidation of both in larger counties relative to smaller ones. Indicative of the social interactions accelerating EV adoption and the barriers to adoption facing lower-income consumers (14).

Indirect network effects further enforce the higher scaling exponents, as limited EVSE infrastructure further depresses EV adoption in smaller counties and accelerates adoption in larger counties (1, 4). Once EVs become widely adopted, we expect registrations to scale linearly with county population (Fig. 1b), driving the scaling of stations with county population (Fig. 1c) towards the sub-linear scaling of stations with registrations (Fig. 1a). Critically, counties with limited infrastructure may lack the critical mass needed to incite widespread EV adoption without intervention (4).

#### Gas station to EVSE scaling

In the long term, as EV adoption increases and fast charging enables a recharging experience comparable to ICE vehicles (3), we expect EVSE stations to tend towards the same scaling exponent as gasoline stations, as both systems provide an analogous utility. However, as the power delivery of an EVSE station is significantly less than that of a gasoline station, we expect that proportionally more stations will be needed to match the utility of existing gasoline stations, as shown in Fig. 1a. Taking the average vehicle miles traveled per capita per unit time *M* and average mileage of *η*, the total power demand for transportation is P∝MN/η. We then assume that *M* remains constant or that consumer demand for transportation is independent of the mode of propulsion used. We can then estimate the number of EVSE stations $Y_{EVSE}$ needed to match the power delivery of $Y_{GS}$ gasoline stations:


(1) $$\frac{Y_{EVSE}}{Y_{GS}}=\frac{\eta_{ICE}P_{GS}}{\eta_{EV}P_{EVSE}}$$

Using the regulated gasoline flow rate of 10 gpm for a consumer pump in the United States (40 CFR §1090.1550) and the EPA’s 33.705 kWh/gallon of gasoline equivalency (40 CFR §600.002), the max power delivery of a consumer gasoline pump is $P_{GS}=20.2$ MW. We assume $\eta_{EV}/\eta_{ICE}\approx 3$, or that, on average, EVs consume 1/3 the energy per mile traveled compared to ICE vehicles. Assuming $P_{EVSE}=400$ kW, or Extreme Fast Charging (3), gives $Y_{EVSE}/Y_{GS}=17$; that is seventeen 400 kW charger ports are required to match the power delivery of one gasoline pump. Reducing $P_{EVSE}$ to 11.5 kW, the upper end of available home chargers, we find $Y_{EVSE}/Y_{GS}\approx 586$. From this, holding the average number of pumps/ports constant, the number of EVSE stations needed, ${\hat{Y}}_{EVSE}$, in an area, is given by Eq. 2.


(2) $${\hat{Y}}_{EVSE}=\frac{1}{3}\frac{P_{GS}}{P_{EV}}\times Y_{0,GS}N^{\beta_{GS}}$$

This adjustment to the scaling pre-factor $Y_{0}$ only accounts for the difference in power delivery between the two infrastructures. Additional factors are required for a complete accounting of their differences. For example, the longer charging time of EVSE stations may require additional stations to prevent excessive wait times. Further, while nearly all ICE vehicles refuel at public gasoline stations, home charging will redirect a proportion of charging demand away from public stations and towards private chargers. Both effects are the result of individual behavior and are thus captured by the pre-factor $Y_{0}$, and not the scaling exponent *β* (10). We can thus model these effects by selecting an appropriate factor *γ* to model the effective demand for EVSE infrastructure: $\gamma {\hat{Y}}_{EVSE}$.

Our present analysis has assumed that EVSE infrastructure will converge on a station design analogous to existing gasoline stations, resulting in $\beta_{EVSE}\approx \beta_{GS}$. However, structural differences could drive EVSEs towards a different scaling exponent than gasoline stations. The power demands of fast-charging stations could drive EVSE stations towards a lower exponent to minimize the length of the electrical network powering the stations (β≈2/3 (7)). Or towards a higher *β*, if the limited power delivery of charging stations inhibits the benefits of agglomeration; and in the extreme case of EVSE infrastructure consisting exclusively of home chargers: β→1. The scaling exponent, $\beta_{EVSE}$, could also deviate if demand for public charging infrastructure or access to private charging (1) varies with population size: $\gamma \propto N^{\delta }$; Resulting in a scaling exponent of $\beta_{EVSE}=\beta_{GS}+\delta$. Here, a δ>0 implies that demand for public charging increases with population reflecting, for example, the reduced availability of home charging in urban counties compared to rural ones. Conversely, δ<0 implies that demand for public charging is higher in rural communities, perhaps due to a higher propensity for longer trips.

### Discussion

Our model (Eq. 2) assumes a fixed average pumps/ports ratio and ignores consumer behavior regarding longer charging times, the role of home chargers, and variations in consumer behavior over time. Our use of $P_{EVSE}=400$ kW effectively assumes that EVSE stations will support a recharging experience similar to gasoline stations (3) and that EVs are a 1:1 replacement for ICE vehicles (15). However, this ($P_{EVSE}$) significantly overestimates the power delivery of current charging stations and EVs (typically less than 150 kW (2, 3)); as such, our model may underestimate the number of stations needed to reach power parity.

Our treatment of existing gasoline stations neglects factors, such as gasoline purchases outside of on-highway transportation, that may inflate the number of stations in a region. Additionally, our model does not account for indirect network effects between EV adoption and EVSE infrastructure or predict the temporal development of charging infrastructure, both key factors for enabling widespread EV adoption (2, 4). As a coarse-grained model, urban scaling does not capture demographic differences between EV adopters and the population at large (1, 14), or its impact on infrastructure development (4). Finally, while scaling laws can capture the coarse-grained regularities over several orders of magnitude, significant deviations remain (Fig. 1). Further refinement is left to optimization-based methods (2), spatial economics (4), or complex systems analysis (5).

Using our expectation of the eventual scaling of EVSE stations (Eq. 2) we estimated the number of stations needed to reach parity with gasoline stations (Fig. 1c); assuming all current and future EVSE stations provide 400 kW chargers. In total, we estimate that 1.8 million stations are required. Notably, we estimate that counties with no EVSE stations (Colored white in Fig. 2), will require ∼270k stations, more than 10x the number of new stations required in Los Angeles County (22k). However, most of these counties are estimated to require fewer than 140 stations. Here, the key insight is that stations per capita decrease with the population of EVs (Fig. 1a); allocating future EVSE stations proportional to population could result in overbuilt urban infrastructure or underserved rural communities. Underserved communities are further impeded by indirect network effects and may lack the critical mass of charging infrastructure needed for widespread adoption (1, 4).

**Fig. 2.**
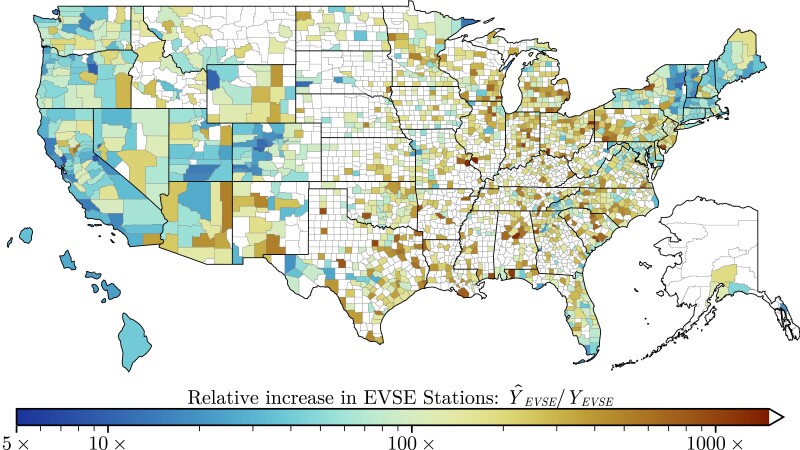
The Scale-Adjusted EVSE Station Gap (${\hat{Y}}_{EVSE}/Y_{EVSE}$) shows the relative increase in the number of existing EVSE stations needed to reach parity with gasoline stations (Eq. 2); assuming all charging occurs at public stations (γ=1) and a power delivery of $P_{EVSE}=400$ kW. This statistic assesses the relative progress of counties toward full electrification while accounting for the expected sub-linear scaling of EVSE infrastructure; resulting in a metric that is independent of population size (12). For example, Platte County, Wyoming, and Allegheny County, Pennsylvania, require a 54-fold expansion of charging infrastructure, despite Platte requiring far fewer stations (∼100 vs. 4.7k). Meanwhile, we predict that Santa Fe County will need a 30-fold expansion in charging infrastructure. An interactive map of our predictions is available online at: https://github.com/BattModels/evse-scaling-behavior.

#### Evaluating existing infrastructure

As shown in Fig. 1c, no county currently has sufficient charging infrastructure to reach parity with gasoline stations; with 1,498 (47.7%) counties lacking any public charging infrastructure. Closing this gap will require orders of magnitude more stations than what presently exists (Fig. 2); with the median county needing a 500-fold expansion. By our metric, Napa County, California, has the most extensive charging infrastructure (8-fold expansion), while St. Clair County, Illinois, has the furthest to go (∼1,400× expansion). A complete tabulation of our county-level predictions is openly available, as described in our data availability section. Due to the present scaling of EVSE infrastructure, smaller counties are more likely to have a greater scale-adjusted EVSE station gap. Sub-linear scaling implies the per capita rates decrease with population size, in stark contrast to the present scaling of charging infrastructure (Fig. 1c). Prioritizing charging infrastructure development using the scale-adjusted EVSE station gap could reduce that discrepancy; helping to ignite EV adoption in presently underserved communities.

## Materials and methods

County-level population estimates were obtained from the United States Census Bureau’s Population Estimates Program using the 2021 Vintage estimates of each county’s population in 2020. We used the fourth quarter 2020 counts for “Gasoline Stations” from the United States Bureau of Labor Statistics Quarterly Census of Employment and Wages Program. EVSE charger locations, and other metadata, were obtained from the National Renewable Laboratory’s Alternative Fuel Stations API and then geocoded to counties using shapefiles obtained from the United States Census. We obtained EV Registrations counts for CA, CO, CT, FL, MI, MN, MT, NK, NY, OR, TN, TX, VA, VT, WA, and WI from the Atlas EV Hub’s State EV Registration data; we were unable to locate data for the remaining 34 states. We performed additional post-processing to account for reporting periods, geographic granularity, missing data, and out-of-state registrations. Passenger Vehicle counts for AK, AL, CA, FL, HI, IA, ID, IL, MD, NE, OH, OK, OR, PA, SD, TX, UT, WA, WI, and WV were obtained from their respective state’s government, primarily from the State’s Department of Motor Vehicles. We were unable to locate publicly available data for the remaining 31 states. As such, our dataset spatially overlaps with both urban and rural areas. We then fit various generalized linear models to the data to predict station counts, EVSE, and Gasoline, for each county using maximum-likelihood estimation as implemented in the Julia Package GLM.jl. For an extended discussion of model fitting, see the Model Fitting section of the Supplementary Material Appendix.

## Supplementary Material

pgad341_Supplementary_Data

## Data Availability

All materials presented here, as well as, a tabulation of our predictions, are openly available for use at https://doi.org/10.5281/zenodo.5784659. Additionally, an interactive map of our analysis is openly available for use at https://github.com/BattModels/evse-scaling-behavior.
