# Supplementary material for: Scaling behavior for electric vehicle chargers and road map to addressing the infrastructure gap
Source: PNAS Nexus. 2023 Oct 25;2(11):pgad341. doi: 10.1093/pnasnexus/pgad341 (PMC10629978; doi:10.1093/pnasnexus/pgad341)
Supplement: pgad341_Supplementary_Data [file pgad341_supplementary_data.pdf]

Supplementary Information for

Scaling Behavior for Electric Vehicle Chargers and Road Map to Addressing the Infrastructure Gap

Alexius Wadell, Matthew Guttenberg, Christopher P. Kempes, and Venkatasubramanian Viswanathan

Venkatasubramanian Viswanathan.
E-mail: venkvis@umich.edu

This PDF file includes:

SI Appendix
SI References
Checklist To Report Theoretical Battery Studies

Contents

1 Data Curation **3**
- A EVSE Stations . . . 3
- B Gasoline Stations . . . 3
- C Vehicle Registrations . . . 3
  - C.1 Alaska . . . 3
  - C.2 California . . . 3
- D Electric Vehicle Registration Counts . . . 4
  - D.1 Mapping ZIP Code Registration data to Counties . . . 4
  - D.2 Out-of-State Registrations . . . 4
  - D.3 Imputing County of Origin . . . 4

2 Model Fitting **4**
- A Generalized Linear Models . . . 5
- B Maximum Likelihood Estimation . . . 5
  - B.1 Poisson Distribution . . . 6
  - B.2 Negative Binomial Distribution . . . 6
- C Ordinary Least Squares . . . 6

3 Statistical Testing **7**
- A McFadden's Pseudo-R Squared . . . 7
- B Root Mean Squared Deviation . . . 7
- C Likelihood Ratio Test . . . 7
- D Parameter Significance . . . 8
- E Compare Parameters . . . 8
- F Bayesian Information Criteria . . . 8

4 Spatial Unit of Analysis **8**
- A Model Fits using Core-Based Statistical Areas . . . 9
  - A.1 Stations vs. Registrations . . . 9
  - A.2 Registrations vs. Population . . . 9
  - A.3 Stations vs. Population . . . 9

Supporting Information Appendix

1. Data Curation

A. EVSE Stations. We tabulated the number of EVSE stations per county using station-level data provided by the National Renewable Laboratory's (NREL) Alternative Fuel Stations API [46]. We then geocoded each station's latitude/longitude coordinates to the bounding county using shapefiles provided by the U.S. Census Bureau [61]. Nearly all stations were reverse geo-coded correctly, except for 7 of the 47,024 stations in the dataset. We manually confirmed the counties for these stations using their street address; in all cases, incorrect coordinates were at fault.

We used the "Open Date" provided by the NREL's dataset to generate time series of EVSE station counts for each county. Effectively we assumed that no station closed or was removed from the dataset. That is, for any date, we assume the number of stations in a county is the number of stations with an opening date before or on that date. For our dataset, we used the number of EVSE stations open on December 31st, 2020 as the 2020 count of EVSE stations in a county.

We estimate the total station power by multiplying the number of Level 1 (1.4 kW), Level 2 (7.2 kW), and DC Fast (50 kW) charging points reported by NREL for each station, by their respective power output shown in parenthesis [2]. Higher power delivery rates are supported by the Tesla Superchargers [58] and the CHAdeMO Protocol 3.0 [15]; however, most EVs are limited to drawing at most 50 kW [2]. Our estimate of the maximum station power delivery may be underestimated, especially for stations that mainly provide DC Fast charging ports.

B. Gasoline Stations. Gasoline station counts at the county level were obtained from the United States Bureau of Labor Statistics (BLS) Quarterly Census of Employment and Wages (QCEW) Program for the fourth quarter of 2020 [60]. We used the number of establishments classified as "Gasoline Stations" under the North American Industry Classification System [28]. This sub-sector includes stations with attached convenience stores (NAICS 447110) and "Other Gasoline Stations" (NAICS 447190). NAICS 447190 includes gasoline stations without attached convenience stores, marine service stations, and truck stops [28]. As such, our count of gasoline stations is inflated beyond the number of stations that service passenger vehicles. This issue also affects the Standard Industrial Classification coding for gasoline stations (SIC 5541) and is no longer used by the BLS for their QCEW program [48].

C. Vehicle Registrations. Vehicle registration counts for 2020 were collected from the following states: AK[4], AL[3], CA[13], FL[21], HI[25], IA[30], ID[27], IL[49], MD[40], NE[47], OH[50], OK[51], OR[52], PA[53], SD[57], TX[59], UT[65], WA[66], WI[70] and WV [68]. We were unable to locate publicly available vehicle registration counts for 2020 for the remaining 31 states. The exact legal definition of a "Passenger vehicle" varies state by state, when available, we preferred registration counts that align with the definition of a "passenger car" as provided by 49 CFR §571.3(c):

> *Passenger car* means a motor vehicle with motive power, except a low-speed vehicle, multipurpose passenger vehicle, motorcycle, or trailer, designed for carrying 10 persons or less.

For example, in Iowa, we used the number of "Automobiles" defined by IA Code §321A.1(42D) as a "motor vehicle designed primarily for carrying nine passengers or less, excluding motorcycles and motorized bicycles." Several states (TX, ID, IL, OK, MD, WV), did not provide a breakdown of registrations by vehicle type, in these cases, we used the total number of vehicle registrations by county. We sought vehicle registration counts representative of the 2020 population counts. We used the 2020 calendar year registration counts for all states but Maryland. Maryland tabulates registration data for the fiscal year; thus, we used the 2020 fiscal year instead of the 2020 calendar year There are additional variations in temporal reporting methodologies from state to state. For example, California reports the number of vehicles registered within the state on January 1st of each year. Conversely, Oklahoma reports the total number of vehicle registrations issued during the calendar year. As such, a vehicle that was registered in California on January 1st, but then moved to Oklahoma within the year, would be double counted by our dataset. Given the rate of domestic migration [12], we suspect the effect of double counting is negligible.

Vehicles registered out of state were excluded from our analysis, even in cases where we could assign the data to particular counties (i.e., California's dataset); as for most states, this was not possible. Additional processing steps were required for Alaska and California, as described below.

C.1. Alaska. Alaska registration counts were provided for governmental boundaries (Boroughs, cities, municipalities) instead of at the county or county-equivalent level. Thankful, we were able to uniquely map all provided governmental boundaries to a single census area, allowing us to tabulate registration counts at the county level. We excluded the 16k passenger vehicles registered in "Other Alaska", as we could not precisely attribute them to any particular county. Additionally, we excluded the 16k passenger vehicles registered to Alaska but residing outside the state, as we had insufficient information to attribute them.

C.2. California. California registration data is tabulated by ZIP Code and not by county, as this analysis requires. We converted ZIP code counts to county-level counts using the US Census's ZIP Code Tabulated Area Crosswalk [63], based on the fractional land overlap between ZIP codes and counties, in line with prior work [20]. Additionally, the California dataset was tabulated by vehicle duty (Light/Heavy). We restricted our dataset to "light-duty" vehicles, as defined by Cal. Code Regs. Tit. 13, §2903, as:

> *Light-duty vehicle* means any passenger car or light-duty truck that meets the applicable definitions in Title 13, section 1900 and is subject to the certification requirements in Title 13, Division 3, Chapter 1, Article 2. A street-use motorcycle is not a light-duty vehicle.

The California dataset additionally breaks out vehicle registrations by fuel type. We considered using this information to attempt to filter out light-duty trucks (i.e., by excluding diesel vehicles). However, we opted against this as diesel passenger vehicles exist [41] and can be served by most gasoline stations.

D. Electric Vehicle Registration Counts. Electric Vehicle registration data for 16 states (CA, CO, CT, FL, MI, MN, MT, NK, NY, OR, TN, TX, VA, VT, WA, and WI) were obtained from the Atlas EV Hub [7]. Registration data was provided at varying intervals (Annually, Bi-Annually, Quarterly or Monthly) and at different levels (ZIP Code or County). We ingested all data as provided and performed the following regularization steps:

1. Map registration data collected at the ZIP code level to counties.
2. Attribute out-of-state registrations to the correct state and resolve differences in reporting intervals.
3. Impute registration county for entries with partial data

Once regularized, we selected each state's latest registration counts for 2020. For example, we used the December count for a state reporting monthly, while for a quarterly reporting state, we used the 4th quarter count. This procedure is consistent with our processing of the EV stations dataset.

D.1. Mapping ZIP Code Registration data to Counties. Consistent with prior works [20], we used the U.S. Census Bureau's ZIP Code Tabulated Area Crosswalk [63] to convert registration data tabulated for ZIP Codes to a county-level tabulation. While most ZIP codes are fully contained within a bounding county, many ZIP codes span multiple counties. As such, the remapping process is a many-to-many join, as multiple ZIP Codes can make up a single county, and a single ZIP code can span numerous counties. We used the fractional area overlap between ZIP codes and counties to remap registrations onto counties.

D.2. Out-of-State Registrations. Out-of-state registrations occur when the location of a vehicle is outside of the state to which it is registered. Out-of-state registrations can arise due to aliasing effects, for example, when a vehicle is registered to a ZIP code spanning state borders. Or due to differences in the residency of a vehicle's owner and the vehicle's primary location. Of the 11.3 million entries in the Atlas EV dataset, 37 thousand (0.33%) are out-of-state registrations. To avoid ignoring these vehicles, we implemented the following procedure:

We deleted the entry for vehicles in states we did not have data for (i.e., a car located in Nevada and registered to California). Including the registration would likely drastically underestimate the number of EVs in the out-of-state county. For vehicles located in a state that we did have data for (i.e., a car located in California and registered in Oregon), we recorded a registration at the date of the registration snapshot. For example, we attribute a vehicle located in California but registered in Colorado to California on the date of the Colorado snapshot; regardless of the reporting interval of California. We then linearly interpolated the registration state's data onto the location state's reporting interval. In the Colorado case, if a CA county had 3 CO vehicles in December and 4 in February, we would attribute 3.5 vehicles for January. However, in practice, CO reports more frequently than CA, eliminating the need for any interpolation. Interpolating out-of-state registration data was solely used to attribute registrations from slowly reporting states (i.e., annually) onto faster reporting intervals (i.e., monthly). We did not use interpolation to increase the reporting rate of states.

We considered using alternative interpolation schemes, such as cubic interpolation or regression, instead of linear interpolation. However, these higher-order methods can suffer from oscillations and can return estimates outside the range of observed values [36, 54]. In most cases, out-of-state registrations are brief; a TX county may have three vehicles registered in CA one month and none the following month. Using linear interpolation ensures we do not overstate the number of vehicles in a county.

D.3. Imputing County of Origin. The county of registration was marked unknown for 28,786 of the 11.3 million (0.25%) entries in the source data set. To avoid listwise deletion, and its associated issues, we used "hot-deck imputation" to impute the missing counties of registration [55, 44, 5]. This is consistent with Myers' recommendation to use hot-deck imputation when the non-response rate is less than 10%. Other more powerful methods, such as Expectation Maximization or Regression Imputation, do not apply to our dataset, as our dataset lacked the requisite data to support these methods. Similarly, mean substitution does not apply to our dataset, as we have data on per-vehicle bias and lack a notion of an "average" country. Imputation was handled at the state level for each reporting snapshot provided to EV Atlas using Impute.jl [29].

In Hot-deck imputation, we replace missing values with "donor" values drawn at random from the rest of the dataset [29, 5]. This procedure preserves the expected fraction of vehicles in a given county. Removing vehicles with an unknown county of origin also preserves the fraction of vehicles in a particular county, but would underestimate the total number of cars.

2. Model Fitting

We had initially sought to leverage urban scaling to model the relationship between refueling/recharging infrastructure and the overall population. Having identified a difference in the scaling for EVSE stations and gasoline stations, we then curated an additional dataset of EV and passenger vehicle registrations. Using our expanded dataset, we then applied urban scaling to model the scaling of vehicle registrations (EV and conventional) with population, and the scaling of recharging/refueling infrastructure with its respective vehicle population.

- Number of EVSE stations vs. county population

- Number of gasoline stations vs. county population
- Number of gasoline stations vs. passenger vehicle registrations
- Number of EVSE stations vs. electric vehicle registrations
- Passenger vehicle registrations vs. county population
- Electric vehicle registrations vs. county population

Building on the procedure developed by Leitao et al., we fit the following models to predict the above relationship [34]:

- Null: $y = c$
- Linear: $y = ax^2 + c$
- Quadratic: $y = ax^2 + bx + c$
- Power (Log-Log Transform): $\ln y = a \ln x + c$
- Power (Log Link): $y = a \ln x + c$

Where "Power (Log-Log Transform)" fits a linear model to the log-log transformed data, similar to prior works [10, 9, 17, 39]. Conversely, "Power (Log-Link)" uses a Generalized Linear Model to predict the log of the expected value ($\ln E[y]$) in line with the recommendation of Leitao et al. [34]. Using a link function instead of log transforming y allows our model to account for $y = 0$, for example, counties with no charging station.

Further, we fit our Generalized Linear Models using the following probability distributions:

- Normal for all models except for "Power (Log Link)"
- Poisson for the "Power (Log Link)" and Null models
- Negative Binomial for the "Power (Log Link)" Null models

A. Generalized Linear Models. Generalized Linear Models (GLM), extend linear regression by introducing a non-linear link function g such that the expected response is: $E[Y|X] = g^{-1}(\eta(X))$, where $\eta(X)$ is the linear function of the dependent variables X. Additionally, GLMs allow distribution of Y to be any distribution $f(Y_i; \theta_i = g(\mu), \phi_i)$ from the exponential family, where θ_i is the natural parameter of the distribution, and ϕ_i is the dispersion parameter of the distribution. We use $i = 1 \ldots n$ to index our n observations and related quantities.

For the power law models, we used the following for g and $\eta(N)$:

$$\begin{aligned} g(\mu) &= \ln \mu \\ \eta(N) &= \ln Y_0 + \beta \ln N \end{aligned} \quad [1]$$

As the expected value of the model $E[Y|N] = g^{-1}(\eta(N))$, this parameterization results in the desired power law model:

$$\mu(N) = g^{-1}(\eta(N)) \rightarrow \exp(\ln Y_0 + \beta \ln N) \rightarrow Y_0 N^\beta$$

B. Maximum Likelihood Estimation. Maximum likelihood estimation (MLE) estimates a model's most probable parameters given the observed data. Resulting in an unbiased estimate of the model parameters, which are the most probable values of the parameters, and in the limit of large sample sizes, approach the true values of the parameters. For a GLM, the likelihood of the model $\mathcal{L}$ is defined by Eq. 2, where f is the probability distribution of the model.

$$\mathcal{L} = \prod_i^n f(Y_i; \theta_i, \phi) \quad [2]$$

$$f(Y_i; \theta_i, \phi) = \exp\left(\frac{Y_i\theta_i - b(\theta_i)}{a(\phi)} + c(Y_i, \phi)\right) \quad [3]$$

For numerical stability, we maximize the log-likelihood ℓ of the model rather than the likelihood $\mathcal{L}$. We used the Julia package GLM.jl [8] to compute the MLE of our model's parameter using the Iteratively Reweighed Least Squares algorithm [26]. The implementation details of the algorithm are beyond the scope of this document.

$$\max \sum_i^n \ln f(Y_i; \theta_i, \phi) \rightarrow \max \sum_i^n \ell_i \quad [4]$$

The log-likelihood of f for a single observation Y_i is given by:

$$\ell_i = \ln f(Y_i; \theta_i, \phi) \rightarrow \frac{Y_i \theta_i - b(\theta_i)}{a(\phi)} + c(Y_i, \phi) \tag{5}$$

The expected value $E[Y_i] = \mu_i$, occurs when $E\left[\partial \ell_i / \partial \theta_i\right] = 0$, thus $\mu_i = b'(\theta_i)$, where b' is the first derivative of b.

$$0 = E\left[\frac{\partial \ell_i}{\partial \theta_i}\right] \rightarrow E\left[\frac{Y_i - b(\theta_i)}{a(\phi)}\right] \rightarrow \frac{E\left[Y_i - b'(\theta_i)\right]}{a(\phi)} \rightarrow E[Y_i] = b'(\theta_i) \tag{6}$$

The canonical link function g of a distribution f is the inverse of b' and thus transforms θ to the mean of the distribution. Alternative link functions are permissible, but the canonical link enables further simplification of the likelihood function, as shown below [1].

B.1. Poisson Distribution. For the Poisson power law models $Y \sim Pois(Y_0 N^{\beta})$, we used the following for $g(\mu)$ and $\eta(N)$:

$$g(\mu) = \ln \mu$$
$$\eta(N) = \ln Y_0 + \beta \ln N$$

Substituting into Eq. 5, for a single observation we get:

$$\ln f(Y_i; \theta_i) = \ln \frac{\theta_i^{Y_i} e^{-\theta_i}}{Y_i!} \rightarrow Y_i \ln \theta_i - \theta_i - \ln(Y_i!) \rightarrow Y_i \cdot \eta(N) - \exp(\eta(N)) - \ln(Y_i!)$$

This gives the criterion for the maximum likelihood estimate for the Poisson power-law models:

$$\underset{Y_0, \beta}{\operatorname{argmax}} \sum_i^n Y_i(\ln Y_0 + \beta \ln N_i) - Y_0 N_i^{\beta}$$

B.2. Negative Binomial Distribution. The negative binomial distribution $NB(Y_i; \theta_i, r)$ has the probability density function given by Eq. 7, where r is a shape parameter, and θ_i is the expected value of the distribution.

$$NB\left(Y_i; \theta_i, r\right) = \frac{\theta^{Y_i}}{Y_i!} \frac{\Gamma(Y_i + r)}{\Gamma(r)(\theta_i + r)^{Y_i}} \frac{1}{(1 + \theta_i / r)^r} \tag{7}$$

This is a transformation of the $NB(Y_i; r, p)$ parameterization, using $\theta = pr/(1-p)$ [33], such that $E[Y] = \theta$ and the variance is $\text{Var}[Y] = \theta\,(1 + \theta/r)$. Additionally, in the limit of $r \rightarrow \infty$, the negative binomial distribution is equivalent to the Poisson distribution. Thus it allows us to extend the Poisson distribution to account for over-dispersion instead of enforcing $\text{Var}(Y) = E[Y]$ [33, 72]. The negative-binomial power-law models $Y \sim NB(\mu = Y_0 N^{\beta}, r)$ using Eq. 1 for the $g(\mu)$ and $\eta(N)$. Substituting Eq. 7, into Eq. 5 gives the log-likelihood for a single observation $(\ln NB(Y_i; \theta_i, \theta))$:

$$Y_i \ln \theta_i - Y_i \ln(\theta_i + r) - r \ln(1 + \theta_i / r) - \ln(Y_i!) + \ln \Gamma(Y_i + r) - \ln \Gamma(r)$$

Dropping terms independent of the parameters (Y_0 and β), and expanding $\theta_i \ = \ln Y_0 + \ln \beta N_i$, gives the following criterion for the maximum likelihood estimate:

$$\underset{Y_0, \beta, r}{\operatorname{argmax}} \sum_i^n \left[Y_i(\ln Y_0 + \beta \ln N_i) - Y_i \ln\left(Y_0 N_i^{\beta} + r\right) - r \ln\left(1 + \frac{Y_0 N_i^{\beta}}{r}\right)\right]$$

As the negative binomial is only a GLM for fixed r, we fit the model using Expectation-Maximization to iteratively alternate between performing MLE of the model parameters (Y_0 and β) and of the shape parameter (r) [8]; until we converge to an MLE estimate for all three parameters. This procedure is consistent with other statistical package [72] and results in an MLE estimate for both the model parameters and the dispersion parameter r [33]. Again, model fitting was performed using GLM.jl [8].

C. Ordinary Least Squares. Prior works in scaling analysis have minimized the least-squares criterion of the log-transformed data [10, 9, 17, 39], as shown in Eq. 8. Expanding Eq. 8 by dropping terms that are independent of Y_0 and β as well as transforming to a maximization problem results in Eq. 9, and is equivalent to a GLM model of the form: $\ln Y \sim \mathcal{N}(\ln Y_0 + \beta \ln N, \sigma^2)$.

$$\begin{aligned} &\underset{Y_0, \beta}{\operatorname{argmin}} \sum_i^n \left[\ln Y_i - (\ln Y_0 + \beta \ln N_i)\right]^2 \rightarrow \\ &\sum_i^n \left[\ln(Y_i)^2 - 2 \ln Y_i \left(\ln Y_0 + \beta \ln N_i\right) + \left(\ln Y_0 + \beta \ln N_i\right)^2\right] \end{aligned} \tag{8}$$

$$\underset{Y_0,\beta}{\text{argmax}}\frac{1}{2}\sum_{i}^{n}\left[\ln Y_i\cdot(\ln Y_0+\beta\ln N_i)-\frac{1}{2}(\ln Y_0+\beta\ln N_i)^2\right] \qquad [9]$$

As noted by Leitao et al., this is an MLE estimate of the model assuming the fluctuations between $\ln Y$ and $\ln Y_0 + \beta$ are normally distributed, and the probability of zero-count data is zero [34]. However, as 43.8% of counties in our dataset have no EVSE stations, $P(y = 0) = 0$ is not a reasonable approximation. For comparison purposes only, we have computed OLS fits of the log-transformed data for both EVSE and gasoline stations but have excluded them from our analysis in favor of models that can fully explain the data.

Fits with Population. For the gasoline station vs. population, our fit had a $R^2_{McF} = 0.903$, a likelihood ratio of $\lambda_{LR} = 30.9 \cdot 10^3$ and a BIC score of $3.35 \cdot 10^3$ based on 3,111 out of 3,143 counties. The fitted scaling exponent was $\beta = 0.77 \pm 0.01$, shows close agreement with previous work [10], and the fitted exponent for the NB model ($\beta = 0.76 \pm 0.01$). These results suggest that approximating the power-law fit with log-transformed data is reasonable for gasoline stations. For the EVSE stations vs. population, our fit had a $R^2_{McF} = 0.754$, a likelihood ratio of $\lambda_{LR} = 12.8 \cdot 10^3$ and a BIC score of $4.20 \cdot 10^3$, based on 1,645 out of 3,143 counties. The fitted scaling exponent was $\beta = 0.71 \pm 0.03$, in contrast to the $\beta = 1.07 \pm 0.05$ predicted by the NB power model. These results suggest that excluding zero-count data significantly alters the model's fit and is inappropriate for the EVSE dataset.

Fits with Registrations. For the gasoline station vs. registrations, our fit had a $R^2_{McF} = 0.859$, a likelihood ratio of $\lambda_{LR} = 12.5 \cdot 10^3$ and a BIC score of $2.08 \cdot 10^3$ based on 1,287 out of 1,288 counties. The fitted scaling exponent was $\beta = 0.66 \pm 0.02$ and shows close agreement with the fitted exponent for the NB model ($\beta = 0.68 \pm 0.02$). For the EVSE stations vs. registrations, our fit had a $R^2_{McF} = 0.81$, a likelihood ratio of $\lambda_{LR} = 5.46 \cdot 10^3$ and a BIC score of $1.34 \cdot 10^3$, based on 498 out of 732 counties. The fitted scaling exponent was $\beta = 0.57 \pm 0.03$, in contrast to the $\beta = 0.70 \pm 0.03$ predicted by the NB power model.

OLS vs. NB Model. We selected the negative binomial version of the power law model for all models presented in the paper, as it directly accounts for zero-count data and thus supports the largest slice of the dataset.

3. Statistical Testing

In order to check the quality of the various GLM fits, perform model selection, and check the significance of the fitted parameters, we performed various statistical tests.

A. McFadden's Pseudo-R Squared. R^2_{McF} compares the log-likelihood of the model ($\ln \mathcal{L}$) to the log-likelihood of the null model ($\ln \mathcal{L}$).

$$R^2_{McF} = 1 - \frac{\ln \mathcal{L}}{\ln \mathcal{L}_0} \qquad [10]$$

To compute $\ln \mathcal{L}_0$, we fit a null model using the same link and distribution but with a constant η and use its log-likelihood for $\ln \mathcal{L}_0$. As noted by McFadden, R^2_{McF} is typically smaller than the R^2 of linear regression, and a value of 0.2 to 0.4 represents an "excellent fit' [42]. Additionally, as R^2_{McF} is a comparison of a model to its null model, it can be used to compare disparate models.

B. Root Mean Squared Deviation. The Root Mean Squared Deviation (RMSD), or Root Mean Squared Error (RMSE), is defined as:

$$\text{RMSD} = \sqrt{\frac{1}{n}\sum_{i}^{n}(\hat{Y}_i - Y_i)^2}$$

Where $\hat{Y}_i$ are the predictions generated by the fitted model, Y_i are the observed values, n is the number of observations, and $i = 1 \dots n$ indexes the observations.

C. Likelihood Ratio Test. The likelihood ratio test can be used to check if the model is significant relative to the null model. For large sample sizes, test statistic λ_{LR} is chi-squared ($\mathcal{X}^2$) distributed under the null hypothesis that the model is not significantly better than the null model [69]. With the alternative hypothesis, that model's fit is significantly different from the null model.

$$\lambda_{LR} = -2(\ln \mathcal{L}_0 - \ln \mathcal{L})$$

We then compare the test statistic to the critical value for $\mathcal{X}^2_k > \lambda_{LR}$ where k, the degrees of freedom for $\mathcal{X}^2$, is the difference in the degrees of freedom between the model and the null model. We found all models to be highly significant with $P < 10^{-99}$ for all models. To compute $\ln \mathcal{L}_0$, we fit a null model using the same link and distribution but with a constant η and use its log-likelihood for $\ln \mathcal{L}_0$.

D. Parameter Significance. To check if the fitted parameters of each GLM model are significantly different from zero, we performed a Wald test to check if the model's parameters are significantly different from zero [67].

$$W = \frac{\bar{X} - \mu}{SE} \quad [11]$$

Where $\bar{X}$ is the value of the fitted parameter, μ is its value under the null hypothesis, and SE is the standard error of the parameter as computed while fitting the model. We then compare the test statistic to the critical value that $W < |\mathcal{N}(0,1)|$. For all fitted models, we found nearly all parameters to be significantly different from zero ($p < 0.001$). For the quadratic model for EVSE count data, we failed to reject the null hypothesis that the intercept parameter was significantly different from zero $p < 0.1$. As the other parameters were significantly different from zero, this does not represent a degenerate model.

In addition, we repeated the test for the power-law fits to check if β was significantly different from one [67]. Using the above test, we found all models at the $p < 0.001$ significance level.

E. Compare Parameters. To compare the fitted coefficients between models, we followed the recommendation of Clogg et. al. and computed the following test statistic [16].

$$z = \frac{\beta_a - \beta_b}{\sqrt{s^2(\beta_a) - s^2(\beta_b)}} \quad [12]$$

Where β_a and β_b are the fitted parameter for the two models respectively; and $s^2(*)$ is the squared standard error for the fitted parameters. We then compare the test statistic z to the standard unit normal $\mathcal{N}(0,1)$. Testing the null hypothesis that the difference between fitted parameters $\beta_a - \beta_b$ is zero against the alternative hypothesis that the difference is significant [16].

We used this statistical test to compare estimates of the scaling parameter β using different spatial units of analysis, as discussed in Section 4.

F. Bayesian Information Criteria. To compare the predictive power of various models, we computed the Bayesian Information Criteria (BIC) for each of the fitted GLMs. The BIC is based on the log-likelihood ($\ln \mathcal{L}$), the number of observations (n) and the number of parameters (k), or complexity, of the model [56].

$$\text{BIC} = k \ln n - 2 \ln \mathcal{L} \quad [13]$$

We can then compare two models by using the BIC scores to estimate the Bayes' Factor B_{12} that one model is better than the other [34, 24].

$$B_{12} \approx \exp\left(\frac{1}{2}\Delta BIC\right) \quad [14]$$

Where $\Delta\text{BIC} = \text{BIC}_2 - \text{BIC}_1$, thus if $\text{BIC}_1 < \text{BIC}_2$ then model 1 is better than model 2; and the likelihood ratio of model 1 vs. 2 is given by Eq. 14. As proposed by Lei et al., we have used $\Delta\text{BIC} > 6$ as our threshold to declare one model is significantly better than the other. This corresponds to a Bayes' Factor of $B_{12} \approx 20.1$ or that one model is at least 20 more likely to describe the data than the other.

4. Spatial Unit of Analysis

Our decision to use counties as the basis of our analysis was driven primarily by data availability, but also our desire to provide estimates for the entire United States. As noted in Section 1, most of our data sources were provided at the county level. As such the use of Urban Area [62] would entail spatially interpolating our dataset and would be highly susceptible to the modifiable unit area problem and other resampling artifacts [6]. We note that prior scaling works have used a variety of spatial units for their analysis including municipalities [35, 32, 14, 34], urban areas [38, 34], metropolitan statistical areas (MSA) [11, 14, 34, 10, 71, 22], core-based statistical areas [37, 23, 22], and commuting zones [43]. Additionally, numerous prior works examining charging infrastructure have similarly used counties as the basis of their analysis [19, 18, 45, 31].

United States Office of Management and Budget does define 929 core-based statistical areas (CBSA) consisting of a "county or counties associated with at least one core . . . plus adjacent counties that have a high degree of social and economic integration with the core measured through commuting ties" [64]. As such, the use of CBSA would not introduce resampling artifacts into our dataset. We note that the Office of Management and Budget cautions against the use of CBSA "as a general-purpose geographic framework" [64]. CBSAs are further divided into Metropolitan Statistical Areas (MSA) and Micropolitan Statistical Areas (µSA), based on the population of the core county. However, data availability and inconsistencies in state-to-state reporting methodologies could introduce significant interpolation and omission artifacts. For example, *New York-Newark-Jersey City, NY-NJ-PA MSA* would be excluded from Fig. 1a and 1b in our manuscript; due to the lack of electric vehicle registration data for NJ and PA. Alternatively, the *Lewiston, ID-WA MSA* spans Washington, which tabulates monthly vehicle registrations by vehicle type, and Idaho, which provides annual reports and does not differentiate vehicle type.

A. Model Fits using Core-Based Statistical Areas. We repeated our model fits using CBSAs instead of counties. To do this, we summed the population, EVSE stations, gasoline stations, EV registrations and vehicle registration counts over the counties composing each CBSA. We excluded CBSAs from analysis if data were missing for any of the constituent counties. For example, the *New York-Newark-Jersey City, NY-NJ-PA MSA* was used when fitting EVSE stations vs. population, as we had all the requisite data, but not for EVSE stations vs. EV registrations as we lack EV registration data for NJ and PA. We followed the same model fitting procedure as described in the Model Fitting Section. We consistently found strong evidence to prefer power-law models over alternative models, as measured by their Bayesian Information Criteria. Using the above statistical tests, we confirmed that the CBSA fits were significant and not degenerative. Additionally, we performed two tests to compare the CBSA estimates of β to our county-based estimates. First, we applied Clogg et. al's recommendation to use a z-test to check if the parameter estimates significantly differed [16]. Second, we checked if the CBSA estimate for β was significantly different from one using a Wald test[67]. The following further discusses our estimates of β using the NB and OLS power law models. Overall, the estimates for β using CBSAs were similar to our estimates using counties. Fig. 2 is a recreation of Fig. 1 using CBSAs, instead of counties, as the spatial unit of analysis.

A.1. Stations vs. Registrations. Both the OLS and NB models provided similar fits for gasoline ($\beta = 0.77 \pm 0.04$ vs. 0.76 ± 0.04) and EVSE ($\beta = 0.68 \pm 0.04$ vs. 0.71 ± 0.04) stations. As before, we find the NB model to be the more appropriate choice, as the OLS model is unable to account for 135 of 392 (34.4%) CBSAs lacking an EVSE station. We were unable to reject the null hypothesis that our CBSA and county-based estimates of β for EVSE stations differed ($P = 0.973$). Additionally, we found evidence that the CBSA estimate was also sub-linear ($P < 0.001$). For gasoline stations, we found evidence that the CBSA and county-based estimates of β differed ($P < 0.001$) and remained sub-linear ($P < 0.001$).

A.2. Registrations vs. Population. We found that OLS models performed significantly better, based on the BIC, the NB model for both gasoline (373 vs. 8996) and EVSE (779 vs. 3841) stations. The OLS model was able to fit all 392 CBSA for which we had data for the gasoline station, but only 275 CBSA, due to zero count data, for the EVSE stations. Both the OLS and NB models provided similar fits for gasoline ($\beta = 1.00 \pm 0.03$ vs. 0.98 ± 0.03) and EVSE ($\beta = 1.43 \pm 0.09$ vs. 1.38 ± 0.11) stations. As before, we find the NB model to be the more appropriate choice, as the OLS model is unable to account for 117 of 392 (29.8%) CBSAs lacking an EVSE station. We were unable to reject the null hypothesis that our CBSA and county-based estimates of β for EVSE stations differed ($P = 0.212$). Additionally, the CBSA estimate of β remained super-linear ($P < 0.001$). For gasoline stations, we failed to reject the null hypothesis that our CBSA and county-based estimates of β differed ($P = 0.621$). Additionally, we failed to reject the null hypothesis $\beta \neq 1$ using our CBSA estimate of β, consistent with our county-based estimate ($P = 0.254$).

A.3. Stations vs. Population. The OLS and NB models provided similar fits for gasoline ($\beta = 0.83 \pm 0.02$ vs. 0.83 ± 0.02) and EVSE ($\beta = 0.95 \pm 0.05$ vs. 1.03 ± 0.07) stations. As before, we find the NB model to be the more appropriate choice, as the OLS model is unable to account for 161 of 927 (17.4%) of CBSAs lacking an EVSE station. Using Clogg's test we failed to reject the null hypothesis that the CBSA and county bases estimates of β for EVSE stations differed ($P = 0.262$). We failed to reject the null hypothesis that $\beta \neq 1$ using a Wald test ($P = 0.407$). We note that our estimate of β remains significantly larger for EVSE than for gasoline stations; consistent with our assessment of EVSE scaling as being out-of-equilibrium. For gasoline stations, we found evidence that the CBSA estimate of β differed ($P < 0.001$) from the county estimate. However, using a Wald test, we found that the estimate remained sub-linear, ($P < 0.001$).

References

[1] Alan Agresti. *Foundations of Linear and Generalized Linear Models*. Wiley Series in Probability and Statistics. Hoboken, New Jersey: John Wiley & Sons Inc, 2015. ISBN: 978-1-118-73005-8 978-1-118-73030-0.

[2] Shabbir Ahmed et al. "Enabling Fast Charging – A Battery Technology Gap Assessment". In: *Journal of Power Sources* 367 (Nov. 2017), pp. 250–262. ISSN: 03787753. DOI: 10.1016/j.jpowsour.2017.06.055. (Visited on 09/27/2021).

[3] Alabama Department of Revenue. *Registration Statistics*. https://www.revenue.alabama.gov/motor-vehicle/registration-statistics/. 2020. (Visited on 08/10/2022).

[4] Alaska Division of Motor Vehicles. *Research & Statistics*. https://doa.alaska.gov/dmv/research/home.htm. 2021. (Visited on 08/10/2022).

[5] Rebecca R. Andridge and Roderick J. A. Little. "A Review of Hot Deck Imputation for Survey Non-response". In: *International Statistical Review* 78.1 (2010), pp. 40–64. ISSN: 1751-5823. DOI: 10.1111/j.1751-5823.2010.00103.x. (Visited on 10/28/2022).

[6] Giuseppe Arbia. *Spatial Data Configuration in Statistical Analysis of Regional Economic and Related Problems*. Ed. by J. P. Ancot and A. J. Hughes Hallet. Vol. 14. Advanced Studies in Theoretical and Applied Econometrics. Dordrecht: Springer Netherlands, 1989. ISBN: 978-94-010-7578-7 978-94-009-2395-9. DOI: 10.1007/978-94-009-2395-9. (Visited on 01/17/2023).

[7] Atlas Public Policy. *State EV Registration Data*. https://www.atlasevhub.com/materials/state-ev-registration-data/. 2021. (Visited on 05/18/2022).

[8] Douglas Bates et al. *JuliaStats/GLM.Jl: V1.5.1*. Zenodo. June 2021. DOI: 10.5281/ZENODO.5044531. (Visited on 11/12/2021).

[9] Luís M. A. Bettencourt. "The Origins of Scaling in Cities". In: *Science* 340.6139 (June 2013), pp. 1438–1441. ISSN: 0036-8075, 1095-9203. DOI: 10.1126/science.1235823. (Visited on 12/02/2021).

[10] Luís M. A. Bettencourt et al. "Growth, Innovation, Scaling, and the Pace of Life in Cities". In: *Proceedings of the National Academy of Sciences* 104.17 (Apr. 2007), pp. 7301–7306. ISSN: 0027-8424, 1091-6490. DOI: 10.1073/pnas.0610172104. (Visited on 05/18/2021).

[11] Luís M. A. Bettencourt et al. "Urban Scaling and Its Deviations: Revealing the Structure of Wealth, Innovation and Crime across Cities". In: *PLoS ONE* 5.11 (Nov. 2010). Ed. by Juan A. Añel, e13541. ISSN: 1932-6203. DOI: 10.1371/journal.pone.0013541. (Visited on 09/18/2021).

[12] US Census Bureau. *State-to-State Migration Flows*. https://www.census.gov/data/tables/time-series/demo/geographic-mobility/state-to-state-migration.html. Oct. 2021. (Visited on 10/27/2022).

[13] California Department of Motor Vehicles. *Vehicle Fuel Type Count by Zip Code - California Open Data*. https://data.ca.gov/dataset/vehicle-fuel-type-count-by-zip-code. May 2022. (Visited on 10/03/2022).

[14] Ben-Hur Francisco Cardoso and Sebastián Gonçalves. *Urban Scaling of COVID-19 Epidemics*. May 2020. DOI: 10.48550/arXiv.2005.07791. arXiv: 2005.07791 [physics, q-bio]. (Visited on 01/11/2023).

[15] CHAdeMO Association. *High Power (ChaoJi) | CHAdeMO*. https://www.chademo.com/technology/high-power. 2022. (Visited on 10/30/2022).

[16] Clifford C. Clogg, Eva Petkova, and Adamantios Haritou. "Statistical Methods for Comparing Regression Coefficients Between Models". In: *American Journal of Sociology* 100.5 (1995), pp. 1261–1293. ISSN: 0002-9602. JSTOR: 2782277. (Visited on 09/28/2022).

[17] Clémentine Cottineau et al. "Defining Urban Clusters to Detect Agglomeration Economies". In: *Environment and Planning B: Urban Analytics and City Science* 46.9 (Nov. 2019), pp. 1611–1626. ISSN: 2399-8083, 2399-8091. DOI: 10.1177/2399808318755146. (Visited on 09/18/2021).

[18] Jairaj Desai et al. "Analysis of Electric and Hybrid Vehicle Usage in Proximity to Charging Infrastructure in Indiana". In: *Journal of Transportation Technologies* 11.4 (Aug. 2021), pp. 577–596. DOI: 10.4236/jtts.2021.114036. (Visited on 01/19/2023).

[19] Jairaj Desai et al. "Using Connected Vehicle Data for Assessing Electric Vehicle Charging Infrastructure Usage and Investment Opportunities". In: *Institute of Transportation Engineers. ITE Journal* 92.3 (Mar. 2022), pp. 22–31. ISSN: 01628178. (Visited on 01/19/2023).

[20] Alexander Din and Ron Wilson. "Crosswalking ZIP Codes to Census Geographies: Geoprocessing the U.S. Department of Housing & Urban Development's ZIP Code Crosswalk Files". In: *Cityscape* 22.1 (2020), pp. 293–314. ISSN: 1936-007X. JSTOR: 26915499. (Visited on 10/03/2022).

[21] Florida Department of Highway Safety and Motor Vehicles. *Vehicle and Vessel Reports and Statistics*. https://www.flhsmv.gov/resources/driver-and-vehicle-reports/vehicle-and-vessel-reports-and-statistics/. 2022. (Visited on 08/10/2022).

[22] Michail Fragkias, José Lobo, and Karen C Seto. "A Comparison of Nighttime Lights Data for Urban Energy Research: Insights from Scaling Analysis in the US System of Cities". In: *Environment and Planning B: Urban Analytics and City Science* 44.6 (Nov. 2017), pp. 1077–1096. ISSN: 2399-8083. DOI: 10.1177/0265813516658477. (Visited on 01/17/2023).

[23] Michail Fragkias et al. "Does Size Matter? Scaling of CO2 Emissions and U.S. Urban Areas". In: *PLOS ONE* 8.6 (June 2013), e64727. ISSN: 1932-6203. DOI: 10.1371/journal.pone.0064727. (Visited on 01/17/2023).

[24] Trevor Hastie, Robert Tibshirani, and Jerome Friedman. *The Elements of Statistical Learning*. 2nd ed. Springer Series in Statistics. New York, NY: Springer New York, 2009. ISBN: 978-0-387-84857-0 978-0-387-84858-7. DOI: 10.1007/978-0-387-84858-7. (Visited on 11/12/2021).

[25] Hawaii State Department of Business, Economic and Development & Tourism's Research and Economic Analysis Division. *The State of Hawaii Data Book 2020*. Tech. rep. Oct. 2021. (Visited on 08/10/2022).

[26] Paul W. Holland and Roy E. Welsch. "Robust Regression Using Iteratively Reweighted Least-Squares". In: *Communications in Statistics - Theory and Methods* 6.9 (Jan. 1977), pp. 813–827. ISSN: 0361-0926, 1532-415X. DOI: 10.1080/03610927708827533. (Visited on 12/13/2021).

[27] Idaho Transportation Department. *Total Vehicle Registrations – 2010 to 2020*. 2022. (Visited on 08/10/2022).

[28] Instituto Nacional de Estadística y Geografía, Statistics Canada, and United States Office of Management and Budget. *North American Industry Classification System*. 2017.

[29] Invenia Technical Computing. *Impute*. Invenia Technical Computing. Oct. 2022. (Visited on 10/28/2022).

[30] Iowa Department of Transportation. *Iowa Fleet Summary By Year, County And Vehicle Type*. https://data.iowa.gov/Transportation-Operations/Iowa-Fleet-Summary-By-Year-County-And-Vehicle-Type/6rrx-2vwt. Oct. 2022. (Visited on 10/27/2022).

[31] Roxana J. Javid, Mahmoud Salari, and Ramina Jahanbakhsh Javid. "Environmental and Economic Impacts of Expanding Electric Vehicle Public Charging Infrastructure in California′s Counties". In: *Transportation Research Part D: Transport and Environment* 77 (Dec. 2019), pp. 320–334. ISSN: 1361-9209. DOI: 10.1016/j.trd.2019.10.017. (Visited on 01/19/2023).

[32] Christian Kühnert, Dirk Helbing, and Geoffrey B. West. "Scaling Laws in Urban Supply Networks". In: *Physica A: Statistical Mechanics and its Applications*. Information and Material Flows in Complex Networks 363.1 (Apr. 2006), pp. 96–103. ISSN: 0378-4371. DOI: 10.1016/j.physa.2006.01.058. (Visited on 05/08/2022).

[33] Jerald F. Lawless. "Negative Binomial and Mixed Poisson Regression". In: *Canadian Journal of Statistics* 15.3 (Sept. 1987), pp. 209–225. ISSN: 03195724, 1708945X. DOI: 10.2307/3314912. (Visited on 11/12/2021).

[34] J. C. Leitao et al. "Is This Scaling Nonlinear?" In: *Royal Society Open Science* 3.7 (July 2016), p. 150649. ISSN: 2054-5703. DOI: 10.1098/rsos.150649. arXiv: 1604.02872. (Visited on 09/18/2021).

[35] Balázs Lengyel et al. "The Role of Geography in the Complex Diffusion of Innovations". In: *Scientific Reports* 10 (Sept. 2020), p. 15065. ISSN: 2045-2322. DOI: 10.1038/s41598-020-72137-w. (Visited on 09/28/2022).

[36] Mathieu Lepot, Jean-Baptiste Aubin, and François H. L. R. Clemens. "Interpolation in Time Series: An Introductive Overview of Existing Methods, Their Performance Criteria and Uncertainty Assessment". In: *Water* 9.10 (Oct. 2017), p. 796. ISSN: 2073-4441. DOI: 10.3390/w9100796. (Visited on 10/28/2022).

[37] José Lobo et al. "Urban Scaling and the Production Function for Cities". In: *PLOS ONE* 8.3 (Mar. 2013), e58407. ISSN: 1932-6203. DOI: 10.1371/journal.pone.0058407. (Visited on 01/17/2023).

[38] Luís M. A. Bettencourt et al. "The Interpretation of Urban Scaling Analysis in Time". In: *Journal of the Royal Society Interface* 17.163 (Feb. 2020), p. 20190846. DOI: 10.1098/rsif.2019.0846. (Visited on 10/11/2022).

[39] Pablo A. Marquet et al. "Scaling and Power-Laws in Ecological Systems". In: *Journal of Experimental Biology* 208.9 (May 2005), pp. 1749–1769. ISSN: 1477-9145, 0022-0949. DOI: 10.1242/jeb.01588. (Visited on 12/15/2021).

[40] Maryland Department of Transportation. *MVA Vehicle Registration by County FY 2010 to FY 2021*. July 2021. (Visited on 08/09/2022).

[41] Matthew Chambers and Rolf Schmitt. *Diesel-Powered Passenger Cars and Light Trucks | Bureau of Transportation Statistics*. Tech. rep. US Department of Transportation, Bureau of Transportation Statistics, Oct. 2015. (Visited on 10/28/2022).

[42] Daniel McFadden. *Quantitative Methods for Analyzing Travel Behaviour on Individuals: Some Recent Developments*. Cowles Foundation Discussion Papers. Cowles Foundation for Research in Economics, Yale University, Nov. 1977, p. 474.

[43] Pricila H. Mullachery and Usama Bilal. "Urban Scaling of Opioid Analgesic Sales in the United States". In: *PLOS ONE* 16.10 (Oct. 2021), e0258526. ISSN: 1932-6203. DOI: 10.1371/journal.pone.0258526. (Visited on 01/17/2023).

[44] Teresa A. Myers. "Goodbye, Listwise Deletion: Presenting Hot Deck Imputation as an Easy and Effective Tool for Handling Missing Data". In: *Communication Methods and Measures* 5.4 (Oct. 2011), pp. 297–310. ISSN: 1931-2458, 1931-2466. DOI: 10.1080/19312458.2011.624490. (Visited on 10/28/2022).

[45] Keisuke Nansai et al. "Life-Cycle Analysis of Charging Infrastructure for Electric Vehicles". In: *Applied Energy* 70.3 (Nov. 2001), pp. 251–265. ISSN: 0306-2619. DOI: 10.1016/S0306-2619(01)00032-0. (Visited on 01/19/2023).

[46] National Renewable Energy Laboratory. *Alternative Fuel Stations API*. 2022.

[47] Nebraska Department of Motor Vehicles. *Annual Registration Statistics by County Categorized by Use Type 2020*. Mar. 2021. (Visited on 10/03/2022).

[48] Occupational Safety & Health Administration of the U.S. Dept. of Labor. *SIC Manual*. 1987. (Visited on 10/30/2022).

[49] Office of the Illinois Secretary of State. *Vehicle Counts by County*. 2021. (Visited on 08/09/2022).

[50] Ohio Department of Public Safety. *Tax Distribution - Detailed Statements of Motor Vehicle Registrations for Year 2020*. 2021. (Visited on 10/03/2022).

[51] Oklahoma Tax Commission. *Annual Vehicle Registration Report*. Tech. rep. 2021.

[52] Oregon Department of Transportation. *Oregon Motor Vehicle Registrations by County*. https://www.oregon.gov/odot/DMV/docs/2020_Vehicle_County_Registration.pdf. 2020. (Visited on 08/11/2022).

[53] Pennsylvania Department of Transportation. *Annual Report of Registrations*. 2021. (Visited on 08/11/2022).

[54] Irfan Pratama et al. "A Review of Missing Values Handling Methods on Time-Series Data". In: *2016 International Conference on Information Technology Systems and Innovation (ICITSI)*. Oct. 2016, pp. 1–6. DOI: 10.1109/ICITSI.2016.7858189.

[55] Philip L. Roth. "Missing Data: A Conceptual Review for Applied Psychologists". In: *Personnel Psychology* 47.3 (1994), pp. 537–560. ISSN: 1744-6570. DOI: 10.1111/j.1744-6570.1994.tb01736.x. (Visited on 10/28/2022).

[56] Gideon Schwarz. "Estimating the Dimension of a Model". In: *The Annals of Statistics* 6.2 (Mar. 1978). ISSN: 0090-5364. DOI: 10.1214/aos/1176344136. (Visited on 11/12/2021).

[57] South Dakota Repartment of Revenue. *Historical Title & Registration Statistics*. Tech. rep. Mar. 2022.

[58] Tesla, Inc. *Supercharger*. https://www.tesla.com/supercharger. 2022. (Visited on 10/30/2022).

[59] Texas Department of Motor Vehicles. *CY 2020 Vehicles Registered and License Fees by County and Regional Office*. Tech. rep. 2021.

[60] U.S. Bureau of Labor Statistics. *Quarterly Census of Employment and Wages*. May 2021. (Visited on 08/16/2021).

[61] U.S. Census Bureau. *2020 TIGER/Line Shapefiles for County and Equivalent*. Feb. 2021. (Visited on 08/16/2021).

[62] United States Census Bureau. *2020 Census Qualifying Urban Areas and Final Criteria Clarifications*. Dec. 2022. (Visited on 01/19/2023).

[63] United States Census Bureau. *2020 ZCTA to County Relationship File*. 2021.

[64] United States Office of Budget and Management. *2020 Standards for Delineating Core Based Statistical Areas*. July 2021. (Visited on 01/11/2023).

[65] Utah State Tax Commission. *UTAH CURRENT REGISTRATIONS 2020*. Tech. rep. 2020.

[66] Washington State Department of Licensing. *Motor Vehicle Registration By Class and County - Calendar 2020*. Tech. rep. 2022.

[67] Larry Wasserman. *All of Statistics: A Concise Course in Statistical Inference*. Springer Texts in Statistics. New York, NY: Springer New York, 2004. ISBN: 978-1-4419-2322-6 978-0-387-21736-9. DOI: 10.1007/978-0-387-21736-9. (Visited on 11/12/2021).

[68] Byrd E White and Everett Frazier. *West Virginia Division of Motor Vehicles Annual Report 2020*. Tech. rep. 2021, p. 36.

[69] S. S. Wilks. "The Large-Sample Distribution of the Likelihood Ratio for Testing Composite Hypotheses". In: *The Annals of Mathematical Statistics* 9.1 (Mar. 1938), pp. 60–62. ISSN: 0003-4851. DOI: 10.1214/aoms/1177732360. (Visited on 11/12/2021).

[70] Wisconsin Department of Motor Vehicles. *Wisconsin DMV Official Government Site - Lists of Vehicle Information.* https://wisconsindot.gov/Pages/about-wisdot/newsroom/statistics/veh-info.aspx. 2022. (Visited on 09/13/2022).

[71] Hyejin Youn et al. "Scaling and Universality in Urban Economic Diversification." In: *Journal of the Royal Society Interface* (2016). DOI: 10.1098/rsif.2015.0937.

[72] Achim Zeileis, Christian Kleiber, and Simon Jackman. "Regression Models for Count Data in *R*". In: *Journal of Statistical Software* 27.8 (2008). ISSN: 1548-7660. DOI: 10.18637/jss.v027.i08. (Visited on 11/12/2021).

Manuscript Title:	Scaling Behavior for Electric Vehicle Chargers and Roadmap to Addressing the Infrastructure Gap
Submitting Author*:	Alexius Wadell

#	Question	Y/N/NA†
1	Have you provided all assumptions, theory, governing equations, initial and boundary conditions, material properties, e.g., open circuit potential (with appropriate precision and literature sources), constant states, e.g., temperature, etc.?	Y
	**Remarks:** Yes, we have provided an in-depth overview of the Generalized Linear Models (GLM) that we used to fit power scaling relations in the supporting information of our paper. We have documented all constants (Pumps Per Station, Efficiency of EVs vs. ICV) and have provided justifications for their inclusion.	
2	If the calculations have a probabilistic component (e.g., Monte Carlo, initial configuration in Molecular Dynamics, etc.), did you provide statistics (mean, standard deviation, confidence interval, etc.) from multiple (≥ 3) runs of a representative case?	NA
	**Remarks:** Calculations, as presented, do not have a probabilistic component. We have provided statistics for our fitted GLM models in the supporting information for our paper.	
3	If data-driven calculations are performed (e.g. Machine Learning), did you specify dataset origin, the rationale behind choosing it, what all information does it contain and the specific portion of it being utilized? Have you described the thought process for choosing a specific modeling paradigm?	Y
	**Remarks:** We have thoroughly documented our sources in the manuscript and supporting information and have made our dataset available online: `https://doi.org/10.5281/zenodo.5784659`	
4	Have you discussed all sources of potential uncertainty, variability, and errors in the modeling results and their impact on quantitative results and qualitative trends? Have you discussed the sensitivity of modeling (and numerical) inputs such as material properties, time step, domain size, neural network architecture, etc. where they are variable or uncertain?	Y
	**Remarks:** We have thoroughly enumerated the possible sources of error in the main text. As a projection of the future infrastructure needs of the United States, our analysis is inherently speculative. However, we have quantified possible sources of uncertainty and explained how our projections could be modified to account for different counterfactual scenarios.	
5	Have you sufficiently discussed new or not widely familiar terminology and descriptors for clarity? Did you use these terms in their appropriate context to avoid misinterpretation? Enumerate these terms in the 'Remarks'.	Y
	**Remarks:** As we sit at the intersection of a few fields (Urban Scaling Analysis, EVCS Infrastructure Modeling), some terminology is likely to be unfamiliar to our readers. We have defined all terms in the main text and have provided citations to relevant literature for further exploration. To the best of our understanding, all terms were used in their appropriate context.<br>In the Main Text: Electric Vehicle Charging Station (EVCS), Electric Vehicle (EV), Internal Combustion Engine (ICE).<br>Expanded upon in the SI: Negative Binomial, Poisson, Bayesian Information Criteria, Generalized Linear Models	

* I verify that this form is completed accurately in agreement with all co-authors, to the best of my knowledge.

† Y ≡ the question is answered completely. Discuss any N or NA response in 'Remarks'.
